# Sitting Posture Monitoring System Based on a Low-Cost Load Cell Using Machine Learning

**DOI:** 10.3390/s18010208

**Published:** 2018-01-12

**Authors:** Jongryun Roh, Hyeong-jun Park, Kwang Jin Lee, Joonho Hyeong, Sayup Kim, Boreom Lee

**Affiliations:** 1 Human Convergence Technology Group, Korea Institute of Industrial Technology, 143 Hanggaulro, Ansan 426-910, Korea; ssaccn@kitech.re.kr (J.R.); freegore@kitech.re.kr (J.H.); sayub@kitech.re.kr (S.K.); 2 Department of Biomedical Science and Engineering (BMSE), Institute of Integrated Technology (IIT), Gwangju Institute of Science and Technology (GIST), Gwangju 61005, Korea; phyeong106@gist.ac.kr (H.-j.P.); lightjin619@gist.ac.kr (K.J.L.)

**Keywords:** sitting posture monitoring system, machine learning, support vector machine, sitting posture classification, load cell

## Abstract

Sitting posture monitoring systems (SPMSs) help assess the posture of a seated person in real-time and improve sitting posture. To date, SPMS studies reported have required many sensors mounted on the backrest plate and seat plate of a chair. The present study, therefore, developed a system that measures a total of six sitting postures including the posture that applied a load to the backrest plate, with four load cells mounted only on the seat plate. Various machine learning algorithms were applied to the body weight ratio measured by the developed SPMS to identify the method that most accurately classified the actual sitting posture of the seated person. After classifying the sitting postures using several classifiers, average and maximum classification rates of 97.20% and 97.94%, respectively, were obtained from nine subjects with a support vector machine using the radial basis function kernel; the results obtained by this classifier showed a statistically significant difference from the results of multiple classifications using other classifiers. The proposed SPMS was able to classify six sitting postures including the posture with loading on the backrest and showed the possibility of classifying the sitting posture even though the number of sensors is reduced.

## 1. Introduction

Ergonomic information provided for the seated person plays a crucial role in improving the sitting posture by changing the habits and attitude of the seated person [1,2,3,4,5]. A study by Robertson et al. [1] reported that musculoskeletal risk was lowered after 16 months by training seated persons with an ergonomic posture. Other studies by Choobineh et al. [2] and Menendez et al. [3] further showed that ergonomic interventions could reduce musculoskeletal symptoms and discomfort. Another study by Taieb-Maimon et al. [5] reported that posture risk was lowered after three weeks in an experiment with a camera showing the sagittal posture of the seated person.

A recent combination of IT technology and various sensors has enabled a sitting posture monitoring system (SPMS) to assess the posture of the seated person in real-time and to improve sitting posture. Previous studies on SPMSs can be classified according to two purposes. The first purpose is to determine whether or not a person is seated on the office chair, which is used mainly to monitor seat occupancy [6,7,8,9]. The second purpose is to detect various sitting postures in order to identify bad sitting postures; this is commonly implemented by inserting pressure sensors into the backrest plate and seat plate [3,8].

Recent advances in machine learning have led to the use of machine learning algorithms in many studies. Machine learning algorithms have the advantage of minimizing errors by training themselves through optimization and tuning and have recently been used in various areas, such as fall detection [10], classification of wrist-motion directions using electromyography [11], and classification of sitting postures [12,13,14,15]. Of these previous studies, the study by Zemp et al. [14] measured data on seven sitting postures using a total of 17 pressure sensors and obtained a maximum classification rate of 90.9% with several classifiers. The study by Meyer et al. [13] developed a textile pressure sensor to measure sitting posture data and obtained a maximum classification rate of 84% using a Naïve Bayes classifier. Furthermore, the study proved that by obtaining data by attaching sensors to the backrest, as well as to the seating plate, the classification was more effective than by attaching sensors to the seating plate exclusively. The study by Zhu et al. [12] measured the data according to the sitting posture on the seat plate and backrest plate using two sensor sheets with 42 × 48 pressure-sensing elements, and further classified the sitting postures using several classifiers. The study by Ma et al. [15] inserted 12 pressure sensors into the seat plate and backrest plate and obtained a maximum classification rate of 99.48% for five sitting postures using several classifiers.

Several studies on sitting posture estimation have proposed SPMSs based on various sensors and have rarely been commercialized because of the high cost of the measuring devices. The prices of products could increase because of the measurement of all loads applied to the sensors that were attached to both the backrest plate and seat plate [7], or by attaching the sensor covering the whole load transfer range [9]. Thus, a new sitting-posture estimation method with a lower cost and fewer device elements is required. In particular, when a new method uses machine learning algorithms, it requires a posture estimation algorithm with higher accuracy according to the individual’s characteristics than the decision tree estimation method using the typical body weight ratio (BWR). This study proposes an algorithm with high posture-estimation accuracy by comparing various machine learning algorithms with a posture estimation method using the decision tree obtained through experiments.

## 2. Methods

### 2.1. Definition of Sitting Posture

**Figure 1:**
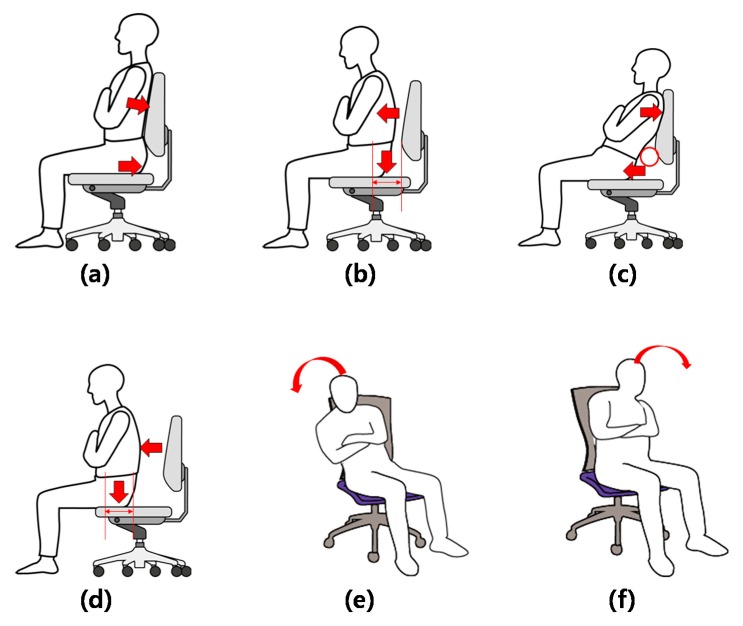
Types of sitting postures adopted for the experiment: (**a**) upright sitting with backrest (UPwB); (**b**) upright sitting without backrest (UPwoB); (**c**) front sitting with backrest (FRwB); (**d**) front sitting without backrest (FRwoB); (**e**) left sitting (LE); and (**f**) right sitting (RI).

Prior to the experiment, the representative sitting posture was defined with reference to previous studies on sitting postures. From these previous studies, the present study derived representative sitting postures, such as the sitting posture in the forward position, middle position, or backward position, forward sitting posture, reclined sitting posture, slumped sitting posture, laterally tilted left or right sitting posture, and crossed legs right over left or left over right sitting posture [6,9,16,17]. This study divided the sitting postures into six types as shown in Figure 1, assuming representative activities in the workplace while sitting on the office chair, with reference to the posture classifications of the previous studies.

### 2.2. System 

**Figure 2:**
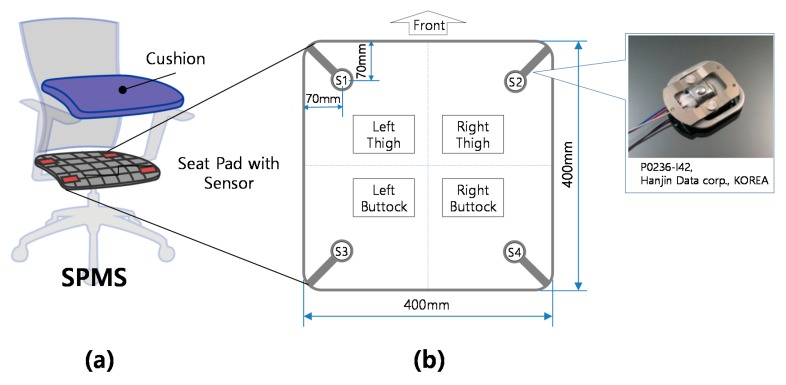
(**a**) Structure of the sitting posture monitoring system (SPMS); and (**b**) arrangement and structure of the pressure sensors in the SPMS.

In this study, an SPMS was fabricated by replacing the seat frame of an existing office chair with a new frame with the load cell inserted (Figure 2). The seat frame of the SPMS had a total of four low-cost load cells (P0236-I42, Hanjin Data Corp., Gimpo, Korea), and the location of each load cell was marked as the left (S1) and right (S2) sides of the thigh position, as well as the left (S3) and right (S4) sides of the buttock position. All the load cells were placed at a distance of 70 mm from each corner of the seat plate. The real-time data on the load (kg) measured on the four load cells were transferred to a personal computer (PC) via the Arduino board. For the main test, the body pressure distribution was measured by placing a body pressure distribution system (Pliance, Novel Corp., Munich, Germany) with the SPMS on the upper part of the seat plate, and two force plates (9260AA, Kistler Corp., Winterthur, Switzerland) were used to measure the loads on the whole chair and the feet. Two webcams (HD-3000, Microsoft Corp., Redmond, WA, USA) were placed in the sagittal and frontal planes of the subject to confirm that the sitting posture was correct.

### 2.3. Procedures

This study involved 24 healthy adult males (age: 27.6 ± 5.6 years, height: 174.5 ± 6.2 cm, and body weight: 71.9 ± 8.7 kg). The selected subjects regularly worked with a video display terminal, sitting on office chairs for eight or more hours a day, and had no apparent severe musculoskeletal deformity or nervous system abnormality. This study was conducted with the approval of the Bioethics Committee of the Public Health Agency designated by the Ministry of Health and Welfare, South Korea (IRB P01-201607-11-001).

The experiment was divided into a preliminary test (15 subjects) and the main test (nine subjects). The 15 subjects were pre-tested to select an SPMS estimation and definition of the BWR range for the sitting posture and in a preliminary test. The other nine subjects participated in the main test for classification using machine learning. For the preliminary test, the load values measured from the load cells of the SPMS were obtained according to the six sitting postures, and the BWR algorithm was defined. Before the experiment, the height of the chair was adjusted to the popliteal height of the subjects, who were fully trained on the classification of the six postures. The subject sat on the office chair according to the instruction on the sitting posture. When the posture was compliant with the instruction, the subject held this posture for 30 s with his arms crossed, and then stood up from the chair. Each posture was repeated five times. For the main test, a total of six sitting postures were randomly changed. The sitting posture was changed every 10 s, and the information regarding each sitting posture was presented with texts and images as well as an auditory signal. After the subject changed the posture according to an auditory signal, the subject maintained the posture with his arms crossed while repeating the procedure. A total of 30 posture changes during each trial were processed from the previous sitting posture to the next sitting posture and derived from the combination of the six sitting postures. The posture change process was designed to avoid an overlap between the previous and succeeding postures. Thus, each trial comprised a total of 30 posture changes over 5 min, and there were a total of three trials (90 posture changes over 15 min). All three trials took place continuously for each of the subjects.

### 2.4. Data Collection

**Figure 3:**
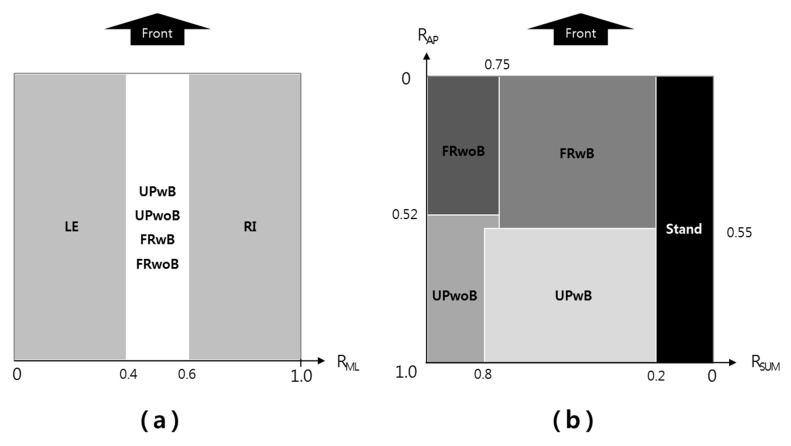
Sitting postures and areas by BWR: (**a**) medial-lateral direction; and (**b**) weight (*X*-axis) plus anterior-posterior direction (*Y*-axis).

The weight (kg) data measured by the four load cells of the SPMS were transferred to a PC at a rate of 1 Hz via the Arduino board. For the preliminary test, the load data collected from the four load cells were classified into a ratio of SPMS to body weight (*R_SUM_*, Equation (1)), a ratio of distribution in the medial-lateral direction (*R_ML_*, Equation (2)), and a ratio of distribution by the anterior-posterior direction (*R_AP_*, Equation (3)). The BWR area determined through the preliminary test is shown in Figure 3. *R_ML_* was used to measure lateral leaning, which was divided into LE, RI, and the other four posture groups (UPwB, UPwoB, FRwB, FRwoB) (Figure 3a). Furthermore, a combination of *R_SUM_* and *R_AP_* resulted in four different posture groups (Figure 3b) according to the load ratio. For the main test, the subjects were trained once on the six postures classified in the preliminary test, and the matching rate between the actual sitting posture and various machine learning algorithms was calculated for the remaining 84 postures, excluding the first six postures. The target posture for the decision tree without machine learning was recorded as the conversion success of the sitting posture only when the conversion posture was kept at the target posture for 5 s or longer.

(1) $$R_{SUM}=\frac{S1+S2+S3+S4}{BW}$$

(2) $$R_{ML}=\frac{S2+S4}{S1+S2+S3+S4}$$

(3) $$R_{AP}=\frac{S3+S4}{S1+S2+S3+S4}$$

where *BW* represents the body weight.

### 2.5. Classifiers

#### 2.5.1. Support Vector Machine

**Figure 4:**
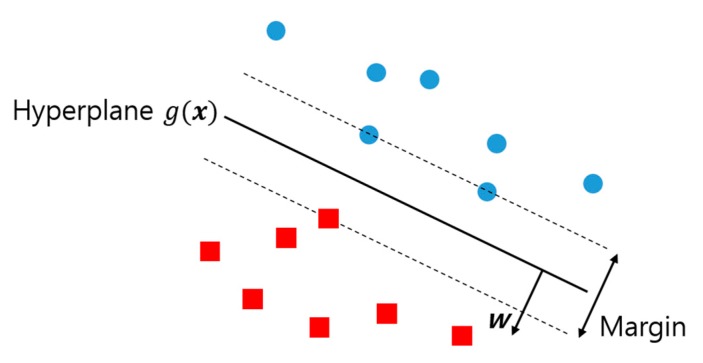
Hyperplane with maximum margins in the linear classifications of two classes (circles and squares).

Support vector machines (SVMs) [18] are the most widely used and one of the highest-performing classifiers because of their high generalization performance [19]. SVMs focus on finding the hyperplane with the maximum margin as shown in Figure 4. The support vector refers to the sample closest to the hyperplane, and the margin refers to the distance between the two support vectors. The hyperplane is typically defined as follows:

(4) $$g\left(x\right)=w^{T}x+w_{0}$$

where *x*, w, and $w_{0}$ represent input vectors, vectors perpendicular to the hyperplane, and constants, respectively. Since the SVM was designed as a binary classifier, the input vector is classified according to the characteristics of the hyperplane as follows:

(5) $$\{\begin{array}{c} g\left(x\right)\geq 1\cdots class\;1\\ g\left(x\right)\leq -1\cdots class\;2 \end{array}$$

The distance between the hyperplane and support vector can be calculated as follows:

(6) $$z=\frac{\left|g\left(x\right)\right|}{||w||}=\frac{1}{||w||}$$

Thus, the margin, the distance between the two support vectors, is represented as follows:

(7) $$\frac{1}{||w||}+\frac{1}{||w||}=\frac{2}{||w||}$$

According to Equation (7) above, the margin becomes maximum when w is minimum. Using the Karush–Kuhn–Tucker condition, a nonlinear optimization equation that minimizes w can be solved. The margin is maximized as follows:

(8) $$\tilde{L}\left(a\right)=\overset{N}{\underset{i=0}{\sum }}\alpha_{i}-\overset{N}{\underset{i=0}{\sum }}\overset{N}{\underset{j=0}{\sum }}\alpha_{i}\alpha_{j}y_{i}y_{j}x_{i}^{T}x_{j}$$

where $\alpha_{i}$ is a Lagrangian multiplier, and the y value has a value of −1 or 1, indicating a class. Applying a kernel function to a SVM can have a great effect on classifying nonlinear data [20]. SVMs with the kernel function applied focus on finding the hyperplane with the maximum margin after transforming the input vector into a higher dimensional space. The kernel function *K* is defined as follows:

(9) $$K\left(x_{i},x_{j}\right)=\Phi {\left(x_{i}\right)}^{T}\Phi \left(x_{j}\right)$$

Using Equation (9), Equation (4) above can be modified as follows:

(10) $$g\left(x\right)=w^{T}\Phi \left(x\right)+w_{0}$$

The kernel function used in this study can be expressed as a radial basis function (RBF) as follows:

(11) $$K\left(x_{i},x_{j}\right)=e^{-\gamma ||x_{i}-x_{j}||{}^{2}},\;\gamma >0$$

#### 2.5.2. Other Classifiers

To verify whether an SVM using the RBF kernel functions best in the system proposed in this study, an SVM using a linear kernel, linear discriminant analysis (LDA), quadratic discriminant analysis (QDA), Naïve Bayes, and a random forest classifier were applied to the data. The LDA is a classification algorithm to find a hyperplane that classifies dependencies by minimizing within-class scatter and maximizing between-class scatter [12,21]. The QDA is a classification algorithm used to find a hyperplane using a quadratic discriminant function when the group covariance matrices are different, as against the LDA that has a constraint in that the group covariance matrices should be the same [22,23]. Naïve Bayes is a probabilistic classification algorithm, assuming conditional independence of predictive attributes [24]. A random forest is a collection of decision trees learned using a random subset of training data [25]. To obtain the classification rate through the learned random forest using test data, the output of each decision tree can be averaged.

**Figure 5:**
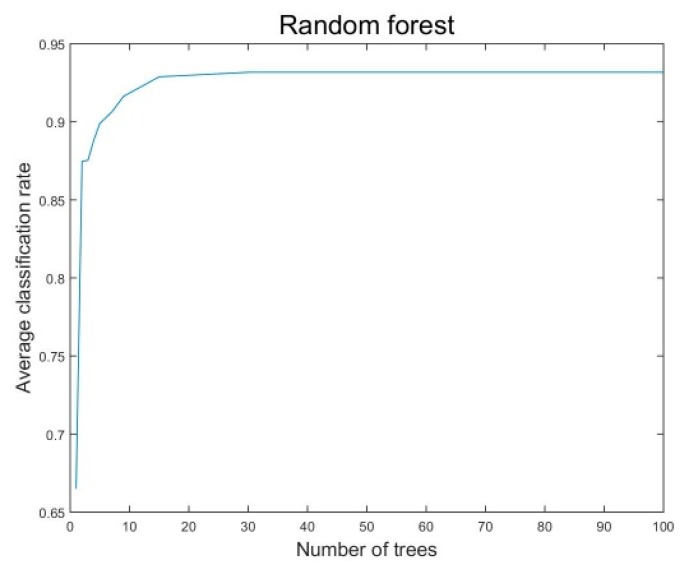
Average classification rate of the random forest classifier according to the number of trees.

A test using the random forest classifier was conducted to determine the number of trees when the classification rate reaches the saturation point. Consequently, we could ascertain the optimal performance with the minimum number of trees. Figure 5 shows that the classification rate reaches the saturation point at approximately 30 trees.

#### 2.5.3. One-Against-All strategy

Since the SVM is a binary classifier, the one-against-all strategy was used to extend the SVM to a multi-classifier. The one-against-all strategy is a method used to create *c* binary classifiers that code the *i* class to 1 and all other classes to −1. In the *c*-class classification problem, the method learns several binary classifiers for multiple classifications by selecting one class and setting all the remaining classes as a different class [26]. The multi-class classification was performed using the one-against-all strategy in all classifiers. The sensor values measured in the load cells were used as the input of each classifier.

## 3. Results

All classifiers were trained using the training data, which contain the six sitting posture changes. The classification accuracy was calculated using the test data, which contain the remaining 84 sitting posture changes. Table 1 shows the classification accuracy of each classifier for the test data; the average classification accuracy ranged from 76.79% to 97.20%. Further, Table 1 shows that the classification rate of the decision tree method was the lowest. The classification rate of the SVM using the RBF kernel ranged from 96.31% to 97.94%. The accuracy of the random forest classifier with 30 trees was the closest to that of the SVM using the RBF kernel, showing only a slight difference. A paired *t*-test was performed to calculate a *p*-value to determine whether there was a statistically significant difference between the results from the SVM using the RBF kernel and those of the other classifiers, and a significant difference was noted (*p* < 0.001).

To determine if the number of sensors used could be further reduced, the sitting position was classified using different classifiers after excluding a specific sensor among the four load cells used. Table 2 shows the average classification rate of each classifier when specific sensors were excluded. As shown in Table 2, no significant difference was noted between the SVM using the RBF kernel and the random forest classifier even with the exclusion of specific sensors. Table 2 further shows that the classification rate did not drop significantly regardless of the exclusion of one or two sensors. However, to determine which sensor was important in classifying the sitting postures, the classification rate for each sensor was measured. Table 2 also shows the average classification rate for each classifier when only one sensor was used. As shown in the last four results in Table 2, the classification rates were higher with S1 and S2 than with S3 and S4, suggesting that sensors S1 and S2 at the thigh position are more important than sensors S3 and S4 at the buttock position.

**Figure 6:**
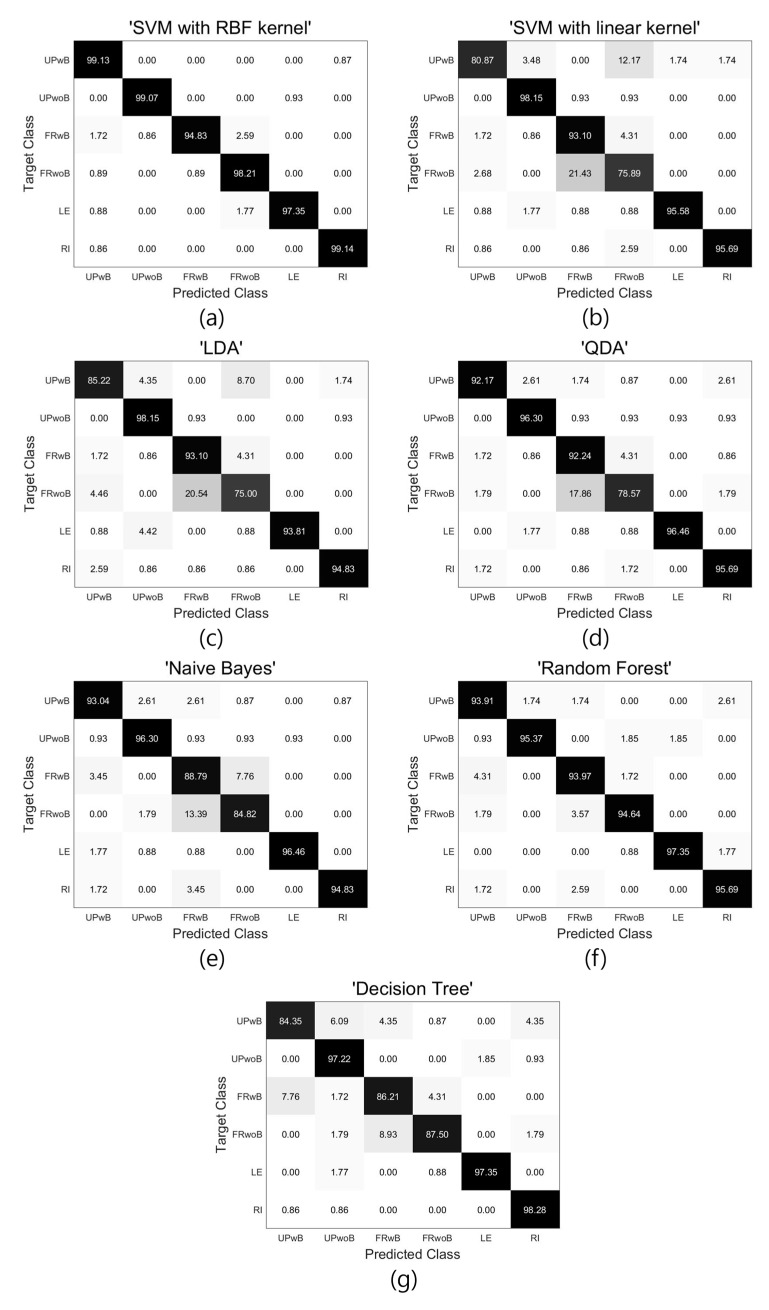
Confusion matrix of the classification results for each classifier in subject 8. (**a**) Support vector machine using the radial basis function kernel; (**b**) support vector machine using the linear kernel; (**c**) linear discriminant analysis; (**d**) quadratic discriminant analysis; (**e**) Naïve Bayes classifier; (**f**) random forest classifier; and (**g**) decision Tree.

Figure 6 shows the confusion matrix for each classification rate in subject 8, which was well classified in all the classifiers. The confusion matrix is a matrix that indicates how many classes in a classification are labeled as true. As shown in Figure 6, most classes for the SVM using the RBF kernel were labeled as true. In the cases of an SVM using the linear kernel, LDA, QDA, and the Naïve Bayes classifier, and FRwoB was frequently classified as FRwB because of the data distribution of the system, with no pressure sensors on the backrest plate, was not linear. Although FRwoB was occasionally classified as FRwB in the random forest classifier, such classification was rare in SVM using the RBF kernel. Figure 6 further shows that the classification error rate regarding the classification of UPwB as FRwoB was high in the cases of the SVM using the linear kernel and LDA. Therefore, classifiers that appropriately classify nonlinear characteristics are suitable for classifying sitting postures.

**Figure 7:**
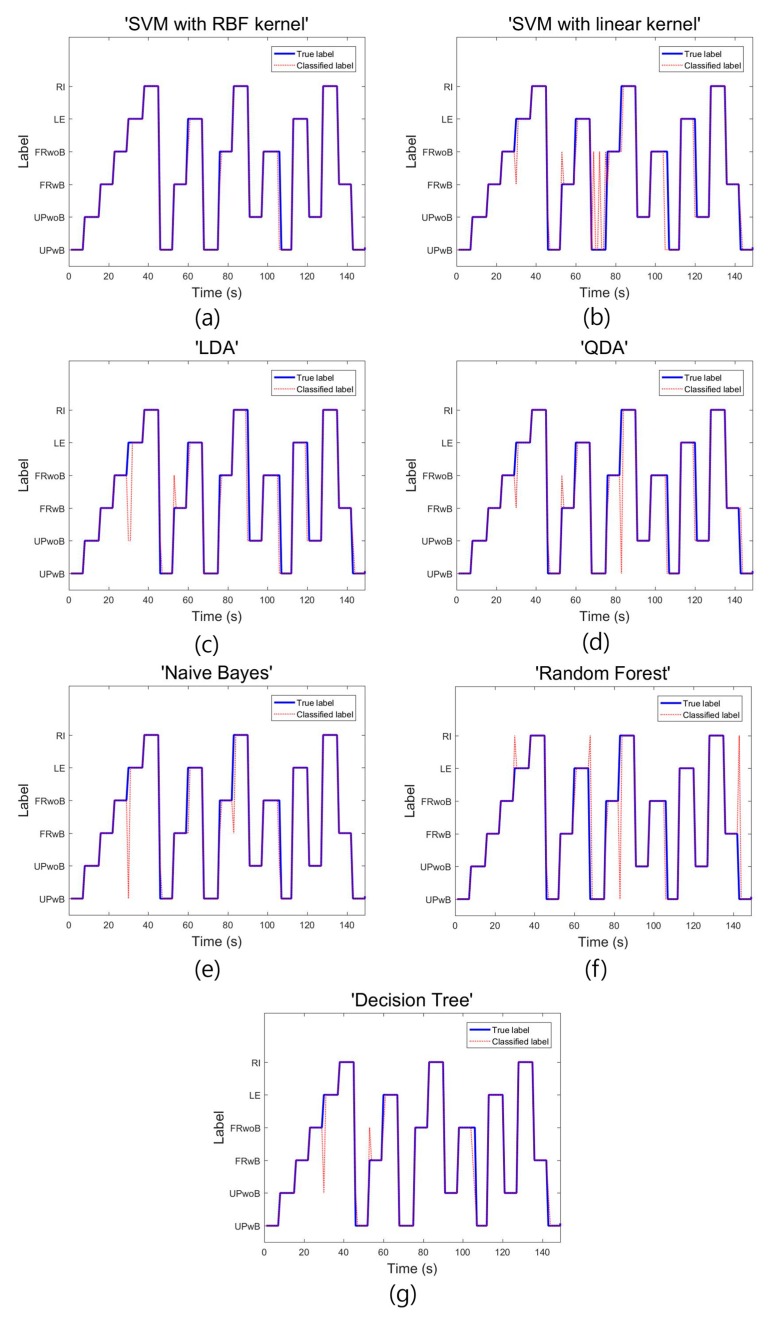
Classification value of the test data for each classifier in subject 8. (**a**) Support vector machine using the radial basis function kernel; (**b**) support vector machine using the linear kernel; (**c**) linear discriminant analysis; (**d**) quadratic discriminant analysis; (**e**) Naïve Bayes classifier; (**f**) random forest classifier; and (**g**) decision tree.

Figure 7 shows the label value of the test data set in subject 8, as well as the result of classifying the test data from each classifier. As shown in Figure 7, in most classifiers, the actual label value differed from the value of the classified label in the intervals where the tasks were changed. This phenomenon could be possible while movement occurs due to the changing tasks and, thus, additional pressure is applied or the pressure on the sensor is weakened. With the SVM using the linear kernel, the data were not well classified in the intervals where the tasks were changed, as well as in the intervals where the tasks were maintained. According to Figure 7, the SVM using the RBF kernel showed a more stable performance than other classifiers even in the intervals where the tasks were changed.

## 4. Discussion and Conclusions

In this study, we proposed a system for monitoring six sitting positions by mounting only four low-cost load cells onto the seat plate of the chair, unlike several studies on the SPMS reported to date, which used several load cells mounted on the seat plate and backrest of a chair. As shown in Table 3, most studies used many more sensors than our proposed system to classify sitting postures. Manli Zhu et al. [12], Zemp et al. [14], and Jan Meyer et al. [13] proposed systems with multiple sensors inserted into the chair to classify various sitting postures, but their classification accuracy was not significantly higher. Congcong Ma et al. [15] used five sensors mounted on both the seat plate and also the backrest of the chair in order to classify five sitting postures with high probability. Using the proposed method, however, we can accurately classify six typical sitting postures using only four sensors exclusively mounted to the seat plate of a chair.

The study used seven classifiers (an SVM using the RBF kernel, an SVM using the linear kernel, LDA, QDA, a Naïve Bayes classifier, a random forest classifier, and a decision tree) to classify the sitting postures via the load cells. The results suggest that the SVM using the RBF kernel is a suitable method for classifying sitting postures with sensors exclusively on the seat plate; the kernel had an average classification rate of 97.20%. Furthermore, the results from the SVM using the RBF kernel showed a statistically significant difference (*p* < 0.05) compared to the results from the other classifiers evaluated.

As shown in Table 2, fewer sensors often resulted in considerable performance degradation, with the exception of the SVM using the RBF kernel and the random forest classifier. Furthermore, Table 2 shows that sensors at the thigh position are more informative than sensors at the buttock position for classifying sitting postures. Based on an analysis of Table 2, the following conclusions can be drawn. In cases where some of the load cells break down or are not activated, the accuracy of sitting posture estimations would indeed be partially reduced, but estimations would nevertheless be possible. Moreover, in terms of commercialization, if the number of load cells must be reduced, the load cells applied to the buttocks (S3 + S4) should be removed before those applied to the thighs (S1 + S2). Figure 6 further shows that the classification of FRwoB as FRwB was very high in the classifiers other than the SVM using the RBF kernel and random forest. The reason for this is that the sensors were attached to the seat plate alone, and the data distribution when sitting in the FRwoB and FRwB positions is the most nonlinear. The performance differences between the classifiers are due to the nonlinear distribution of the sitting posture data, which suggests that the SVM using the nonlinear RBF kernel could outperform the other classifiers. Moreover, as shown in Figure 7, although most classifiers failed to classify the data in the intervals where the sitting postures were changed, the SVM using the RBF kernel better classified the data than the other classifiers. The results show that the SVM using the RBF kernel is more robust compared to the other classifiers, and is more suitable for classifying sitting postures in systems where sensors are attached to the seat plate alone.

In this study, we developed a system that classifies six sitting postures using four load cells mounted only onto the seat plate of the chair and obtained high classification accuracy. However, we did not apply our system in real-time. Future studies shall apply our method to analyze sitting postures in real-time by integrating a field-programmable gate array (FPGA), which can improve the computation time and power consumption of the hardware in order to classify sitting postures in real-world settings. Moreover, in this study, environmental variables were limited in order to maximize the accuracy of the system. Future studies shall explore variations in posture (e.g., the use of armrests, changes of foot position, the height of the seat, etc.). Finally, as shown in Table 2, future studies will explore the optimal sensor position in order to construct an SPMS with fewer sensors, and develop a feature selection algorithm and a classification algorithm that improves the classification rate with fewer sensors.

## Figures and Tables

**Table 1:** Classification rate of test data according to classifier in each subject.

Subject	$SVM_{rbf}$	$SVM_{lin}$	*LDA*	*QDA*	*NB*	*RF*	*DT*
1	**0.9736**	0.7850	0.8197	0.8502	0.7642	0.9182	0.6089
2	**0.9777**	0.8579	0.8830	0.8774	0.7618	0.9443	0.7145
3	**0.9678**	0.8860	0.9020	0.9035	0.9006	0.9327	0.8728
4	**0.9722**	0.8944	0.9042	0.9042	0.8806	0.9361	0.8125
5	**0.9721**	0.8830	0.9164	0.9206	0.8733	0.9415	0.7604
6	**0.9624**	0.8790	0.8887	0.8915	0.7928	0.9263	0.6787
7	**0.9721**	0.8607	0.9011	0.9248	0.8482	0.9248	0.8301
8	**0.9794**	0.8971	0.9000	0.9191	0.9235	0.9515	0.9176
9	**0.9705**	0.8215	0.8555	0.8687	0.8451	0.9100	0.7153
Average	**0.9720**	0.8627	0.8856	0.8956	0.8433	0.9317	0.7679
*p*-value		p<0.001	p<0.001	p<0.001	p<0.001	p<0.001	p<0.001

**Table 2:** Average classification rate of test data according to classifier, including specific sensors.

Included Sensor	$SVM_{rbf}$	$SVM_{lin}$	*LDA*	*QDA*	*NB*	*RF*
S1, S2, S3, S4	0.9720	0.8627	0.8856 *	0.8956	0.8433	0.9317
S1, S2, S3	0.9621 **	0.8367	0.8693 *	0.8858	0.8306	0.9250 *
S1, S2, S4	0.9655	0.8193 *	0.8463 *	0.8720	0.8235	0.9250 *
S1, S3, S4	0.9684	0.7844 *	0.8031 *	0.8594 *	0.8120 *	0.9232 **
S2, S3, S4	0.9632 *	0.8174	0.8440 **	0.8744	0.8272	0.9219 *
S1, S2	0.9280 **	0.6528 **	0.6897 **	0.7514 **	0.7525 **	0.8943 **
S1, S3	0.9388 **	0.6754 **	0.7165 **	0.8020 **	0.8037 *	0.9048 **
S1, S4	0.9413 **	0.6333 **	0.7315 **	0.8021 *	0.7226 **	0.9060 **
S2, S3	0.9417 **	0.6780 *	0.7199 **	0.8105 *	0.7594 *	0.8973 *
S2, S4	0.9452 **	0.6733 **	0.7649 **	0.8305 *	0.8130 *	0.9114 *
S3, S4	0.9372 **	0.6105 *	0.6886 **	0.7352 **	0.7058 *	0.8851 **
S1	0.8435 **	0.3795 **	0.5104 **	0.6420 **	0.6420 **	0.7864 **
S2	0.8467 **	0.4281 **	0.5285 **	0.6540 **	0.6540 **	0.7922 **
S3	0.8090 **	0.2495 **	0.4710 **	0.5547 **	0.5547 **	0.7174 **
S4	0.8177 **	0.2778 **	0.4837 **	0.5462 **	0.5462 **	0.7265 **

* represents p<0.05 which compared with the results of using whole sensors. ** represents p<0.001 which compared with the results of using whole sensors.

**Table 3:** Comparison between the previous studies and proposed system.

Author	Number of Sensors	Location of Sensors	Number of Subjects	Classification Algorithm	Number of Posture	Classification Accuracy
Manli Zhu et al. [12]	**Two pressure sensor sheets (42 × 48 pressure sensor)**	Seat plate and backrest	50	Slide inverse Regression	10	86%
Zemp et al. [14]	**16 pressure sensors**	Seat plate, backrest, and armrest	41	Random Forest	7	90.9%
Jan Meyer et al. [13]	**96 pressure sensors**	Seat plate	9	Naïve Bayes	16	82%
Congcong Ma et al. [15]	**12~5 pressure sensors**	Seat plate and backrest	11	j48 decision tree	5	99.51%
Proposed method	**4 load cells**	Seat plate	9	SVM using RBF kernel	6	97.20%

## Abbreviations

The following abbreviations are used in this manuscript:

SPMS	Sitting posture monitoring system
RBF	Radial basis function
BWR	Body weight ratio
UPwB	Upright sitting with backrest
UPwoB	Upright sitting without backrest
FRwB	Front sitting with backrest
FRwoB	Front sitting without backrest
LE	Left sitting
RI	Right sitting
PC	Personal computer
R_SUM_	Ratio of SPMS to body weight
R_ML_	Ratio of distribution in the medial-lateral direction
R_AP_	Ratio of distribution by the anterior-posterior direction
SVM	Support vector machine
LDA	Linear discriminant analysis
QDA	Quadratic discriminant analysis
NB	Naïve Bayes
RF	Random forest
DT	Decision tree
